# Simultaneous and preceding sounds enhance rapid visual targets: Evidence from the attentional blink

**DOI:** 10.2478/v10053-008-0139-4

**Published:** 2013-09-20

**Authors:** Cornelia Kranczioch, Jeremy D. Thorne

**Affiliations:** 1 Neuropsychology Lab, Department of Psychology, Carl von Ossietzky University Oldenburg, Germany; 2 Research Center Neurosensory Science, Carl von Ossietzky University Oldenburg, Germany

**Keywords:** attentional blink, sound, tone, practice, crossmodal facilitation, reliability

## Abstract

Presenting two targets in a rapid visual stream will frequently result in the second target (T2) being missed when presented shortly after the first target (T1). This so-called *attentional blink* (AB) phenomenon can be reduced by various experimental manipulations. This study investigated the effect of combining T2 with a non-specific sound, played either simultaneously with T2 or preceding T2 by a fixed latency. The reliability of the observed effects and their correlation with potential predictors were studied. The tone significantly improved T2 identification rates regardless of tone condition and of the delay between targets, suggesting that the crossmodal facilitation of T2 identification is not limited to visual-perceptual enhancement. For the simultaneous condition, an additional time-on-task effect was observed in form of a reduction of the AB that occurred within an experimental session. Thus, audition-driven enhancement of visual perception may need some time for its full potential to evolve. Split-half and test-retest reliability were found consistently only for a condition without additional sound. AB magnitude obtained in this condition was related to AB magnitudes obtained in both sound conditions. Self-reported distractibility and performance in tests of divided attention and of cognitive flexibility correlated with the AB magnitudes of a subset but never all conditions under study. Reliability and correlation results suggest that not only dispositional abilities but also state factors exert an influence on AB magnitude. These findings extend earlier work on audition-driven enhancement of target identification in the AB and on the reliability and behavioural correlates of the AB.

## Introduction

A multitude of studies exist demonstrating conditions in which we fail to consciously perceive stimuli or events that are clearly above threshold. One such phenomenon is the attentional blink (AB). The AB is a deficit in detecting the second of two targets (T1, T2) that are presented within a rapid serial visual presentation (RSVP) of non-targets or distracters (Raymond, Shapiro, & Arnell, 1992). In a typical AB paradigm, stimuli are presented at a rate of 10 per second, and the window during which the deficit is observed lasts for about half a second. The deficit is usually largest when T2 is presented at Lags 2 or 3, that is, as the second or third stimulus after T1. Many studies report that the deficit spares T2 when presented directly after T1 with no intervening non-target, which is referred to as *Lag 1 sparing*. Despite considerable differences in the theories of the AB (for a review, see Dux & Marois, 2009), they all have in common the assumption that perceptual processing of T2 is initially unimpaired, but that the perceptual representation of T2 is at risk of being masked or of decaying before it can be selected for further processing.

Olivers and Van der Burg (2008) reported that a sound presented simultaneously with T2, but carrying no information with regard to the identity of T2, improved T2 identification both at short and long T2 lags. Descriptively, this effect was larger for T2 stimuli presented at Lag 2 than for those presented at Lag 5, though this difference did not reach significance. In contrast, a sound that preceded the target by 250 ms had little if any effect.^1^ These findings replicated earlier work by Vroomen and de Gelder (2000), who reported that a simultaneous non-specific auditory cue could improve the detection of a single target pattern embedded in an RSVP stream of non-target patterns. Following Vroomen and de Gelder’s interpretation, Olivers and Van der Burg concluded that the effect of the sound is mostly automatic and perceptual in nature. That is, they suggested that the sound boosts the visual representation of T2, which is therefore more likely to escape the AB. A recent study by Ngo and Spence (2010) confirmed the enhancing effect of a simultaneous sound on the identification of a single visual target observed by Vroomen and de Gelder (2000). It also showed that the effect is not restricted to auditory cues but can be observed for visual and tactile cues as well. An alerting condition was not tested.

To date the study by Olivers and Van der Burg (2008) is the only study on healthy volunteers that has investigated cross-modal reduction of the AB, that is, the impairment in identifying a target that follows shortly after another target. This seems surprising, given that the AB itself is a widely studied phenomenon, and given empirical evidence suggesting that the AB is influenced by many factors and thus reflects a different deficit compared with the problems that arise when identifying a single target or the first of two successive targets in a rapid stimulus stream (Ambinder & Lleras, 2009). Moreover, research employing the AB paradigm does not provide unequivocal support for the findings of Olivers and Van der Burg. In detail, a recent study reports that visual cues that precede T2 can improve its identification (Spalek & Di Lollo, 2011), and in a study by Van Vleet and Robertson (2006), a sound that preceded T2 was found to improve T2 performance in a patient suffering from visuo-spatial neglect. Even though these studies did use an AB paradigm, they differed in many aspects from the study by Olivers and Van der Burg. Thus, a large number of factors could potentially have contributed to the discrepancy in the findings. But irrespective of that, one fundamental question that remains is whether the findings of cross-modal facilitation of T2 identification by a simultaneous tone and concomitant non-facilitation by a preceding tone in the AB paradigm (as initially reported by Olivers and Van der Burg) can be replicated. To address this basic question, we studied T2 identification performance in three versions of the AB task: T2 was presented in isolation, T2 was presented simultaneously with a tone, or T2 was preceded by a tone. We expected to replicate the T2-related findings by Olivers and Van der Burg, namely that the simultaneous tone improves T2 identification as compared to when no tone is presented, whereas the preceding tone should not improve performance. The replication of the T2 findings of Olivers and Van der Burg would be an indicator of their reliability.

The present study was additionally aimed at the investigation of classical measures of reliability of the AB effect in the conditions under study, an important step in the further understanding of the AB’s underlying mechanisms (Dale & Arnell, 2011; Kelly & Dux, 2011). The study was furthermore expected to contribute to the understanding of the effects or non-effects of cross-modal cuing on the AB. Previous research on AB paradigms not containing an additional tone suggests that the AB deficit is reliable in terms of split-half reliability or test-retest reliability (Dale & Arnell, 2011; Kelly & Dux, 2011). Alterations in the details of the AB paradigm might however largely reduce reliability (Kelly & Dux, 2011), though evidence here is mixed (Dale & Arnell, 2011). In the present study, the reliability of the AB was assessed by means of test-retest and split-half reliability, and by comparing performance across conditions. An additional question in the context of reliability was whether AB magnitudes obtained in the three conditions might have comparable predictors. This would be expected if each reflected mostly the same underlying deficit. To this end we explored correlations with measures of attentional performance, in particular, the ability to divide attention between an auditory and a visual task and the ability to integrate auditory and visual information. Moreover, research by MacLean and Arnell (2010) suggests that cognitive flexibility relates to T2 performance and AB magnitude. Cognitive flexibility was therefore measured as a further variable in the present study. Finally, results by Forster and Lavie (2007) suggest that everyday distractibility can be related to the experienced degree of interference from irrelevant distracters in laboratory tasks. For the AB task versions studied here, the tone might be regarded as an additional distracter. We therefore asked whether everyday distractibility would be related to whether participants did or did not benefit from the tone.

## Material and methods

### Subjects

The local ethics committee approved the study in which 24 paid participants (seven male, 17 female) took part. Three participants were excluded from the sample for reasons described in detail below. The final sample of 21 participants (seven male, 14 female) had a mean age of 23 years (range 19-28 years). All participants were right handed, free of current or past neurological or psychiatric illness, had normal or corrected-to-normal vision, normal hearing, and gave informed consent prior to participation.

### Stimuli and procedure

Behavioural data were collected in the context of a larger study, which focused on the neural correlates of conscious visual perception and its modifiability by auditory input. The study was designed such that it comprised three sessions. In Sessions 1 and 3, the AB experiment was performed. In addition to behavioural data, electroencephalogram (EEG) data were collected during the AB experiment and during a resting period. EEG data are beyond the current scope and will therefore not be reported here. In Session 2, participants answered two questionnaires. The first was a German version of the Cognitive Failures Questionnaire (CFQ; Broadbent, Cooper, FitzGerald, & Parkes, 1982; Lumb, 1995), and the second was the NEO-Fünf-Faktoren-Inventar (NEO-FFI), a German 60-item scale measuring the five domains of the Revised NEO Personality Inventory (NEO-PI-R; Costa & McCrae, 1992). In the NEO-FFI, each trait (neuroticism, extraversion, openness, conscientiousness, and agreeableness) is assessed with 12 items. Additionally, participants performed some neuropsychological attention tests for which subtests of the Test of Attentional Performance (TAP; Zimmermann & Fimm, 2007) were used. The TAP is a computer-based test battery of basic attentional performance. The subtests Crossmodal Integration, Divided Attention I, and Flexibility (verbal) were used. The subtest Crossmodal Integration tests the abili-ty to integrate a visual and an auditory stimulus. A high or low tone precedes a visual stimulus, which is an arrow pointing up or down. The critical combinations requiring a response are a high tone precedingan arrow pointing up and a low tone preceding an arrow pointing down. The Divided Attention I subtest tests how well participants are able to attend to an auditory and to a visual sequence of events simulta-neously. Participants are first presented with a visual, then an auditory sequence of events. A button needs to be pressed in response to the appearance of pre-defined targets. In the third run, visual and auditory sequences are presented simultaneously, and subjects are instructed to press the button if they detect a visual or an auditory target. The subtest Flexibility is a set shifting task, measuring the ability to flexibly orientone’s attentional focus. A letter and a number are simultaneously presented to the right and the left of the centre of the monitor. The target stimulus (letter or number) alternates from trial to trial, and the participant’s task is to press a left or right key according to where the current target stimulus appears on the monitor. For all participants, the interval between Sessions 1 and 3 was 14 days. The interval between Session 1 and Session 2 was on average 7.1 days (range 6 to 8 days).

**Figure 1.**
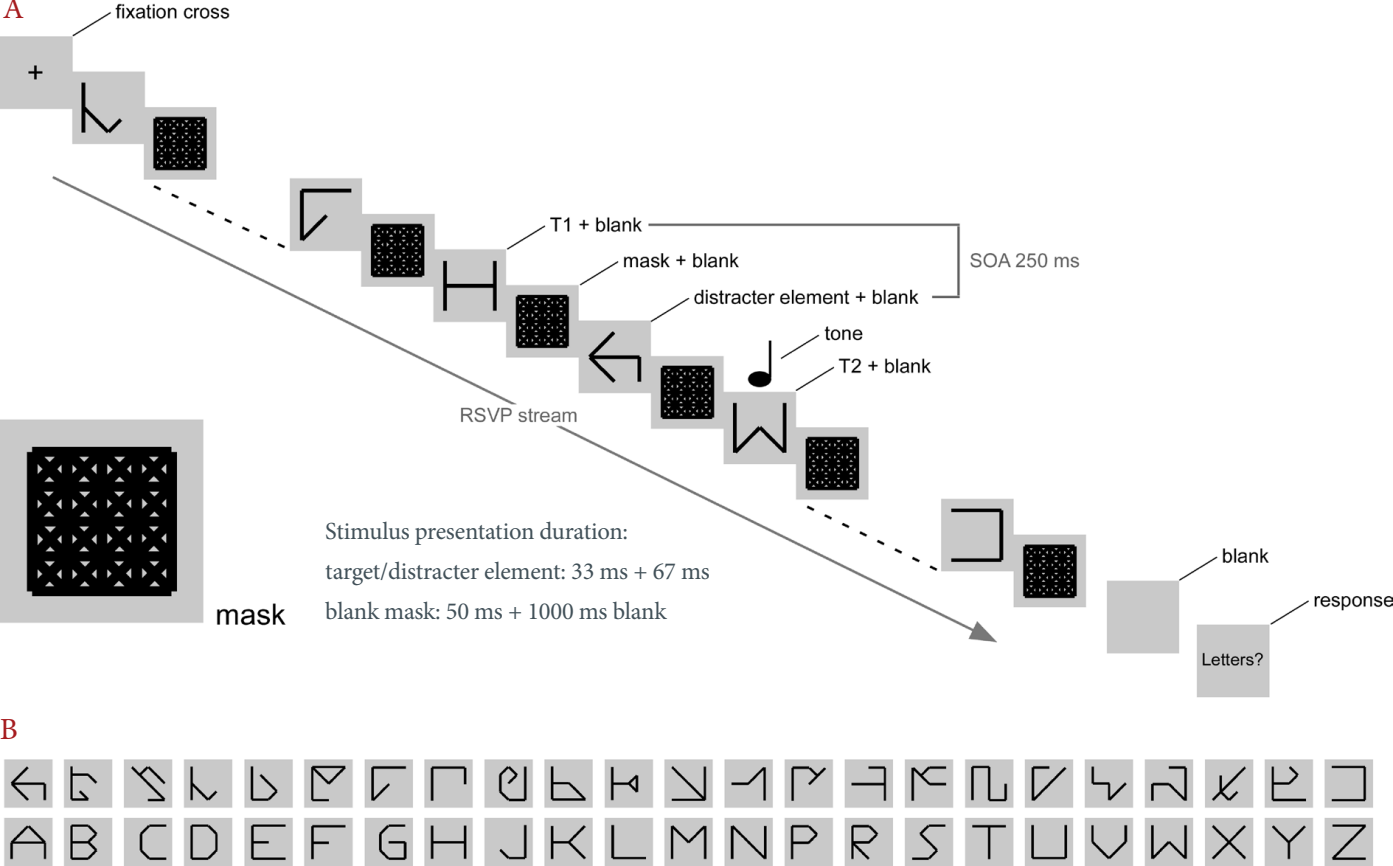
Panel A. Trial layout. The illustration shows a trial in which T2 was presented at Lag 2 and in which a tone was played simultaneously with T2. Distracter elements and target elements (T1, T2) were displayed for 33 ms. Elements were followed by a mask at a stimulus onset asynchrony (SOA) of 100 ms. Masks were displayed for 50 ms and also followed by a blank screen, resulting in a target-to-distracter SOA of 250 ms and a target-to-target SOA of 500 ms (Lag 2) or 1,250 (Lag 5). A trial could contain no tone or the second target (T2) was either preceded by 250 ms or accompanied by a tone. The rapid serial visual presentation (RSVP) stream was preceded by a fixation cross and followed first by a blank screen and then by a response screen. Responses were un-speeded. Bottom left: Enlarged mask stimulus. Panel B. Illustration of all used distracter (top row) and target (bottom row) stimuli.

Participants were seated comfortably in a sound-attenuated, dimly-lit booth. Stimuli were presented using Presentation 14.5 (NBS Inc.) software. The computer monitor was placed outside the booth at a distance of approximately 175 cm from the participant. Monitor refresh rate was 60 Hz. The RSVP stream consisted of distracter elements, target elements, and masks similar to those used by Olivers and Van der Burg (2008): Target elements were letters (except I, O, Q), distracter elements were meaningless shapes, masks were patterned squares (cf. Figure 1). All stimuli were designed based on a square that consisted of 16 smaller squares, arranged in a 4 × 4 array. The complete square served as the mask, and letters and scrambled letters were built from a selection of line segments within the square (see Figure 1). Stimulus size was 1.87 by 1.87 degrees of visual angle for the mask, 1.4-1.87 by 1.87 degrees of visual angle for target and distracter elements, and 0.33 degrees of visual angle for the fixation cross that preceded the RSVP stream. All stimuli were black and were presented centrally on a grey background.

Distracter and target elements were presented for 33 ms, followed by 67 ms of blank screen.^2^ Between two distracters or between a target and a distracter the mask was presented for 50 ms, again followed by a blank screen of 100 ms duration. This resulted in a target-to-target stimulus-onset asynchrony (SOA) of 500 ms for the Lag 2 (or short target-to-target SOA condition) and of 1,250 ms for the Lag 5 (or long target-to-target SOA condition). The tone could be played simultaneously with the pre-T2 distracter (SOA tone-T2 = 250 ms) or simultaneously with T2 (SOA tone-T2 = 0 ms). This design was chosen in accordanceto Olivers and Van der Burg (2008), and was based on evidence indicating that audiovisual binding is rather bad with a 250-ms SOA (e.g., Fujisaki & Nishida, 2005). That is, if distracter-to-distracter or distracter-to-target SOA were around 100 ms as in typical AB studies, participants would frequently fail to bind the sound to the simultaneous stimulus and instead would bind it to the preceding or subsequent stimulus. Note that this design results in distracter/target SOAs substantially longer than the approximately 100 ms applied in typical AB paradigms. In consequence, the Lag 2 T2 is presented at a time where in a typical AB paradigm the AB has largely recovered. Although one could thus argue that the present design is not suited to study the AB, Olivers and Van der Burg showed that in spite of the longer SOAs T2 identification, performance is substantially impaired at short T2 lags as compared to long T2 lags. This is seen as the primary indicator of the AB effect (MacLean & Arnell, 2012).

The auditory stimulus was a 700 Hz tone played for 32 ms via speakers at comfortable loudness. Loudness was the same for all participants and both sessions, and it was assured before the start of the experiment that none of the participants perceived the auditory stimulus as too quiet or too loud. The tone included a 5-ms fade-in and fade-out time to avoid clicks. Stimulus timing of audio-visual stimulus pairs was verified by an external testing device (Cedrus Corporation, San Pedro, USA).

Each trial started with the presentation of a black fixation cross for 1,000 ms, which was followed by the RSVP stream. The RSVP stream consisted of alternate presentations of distracter elements and masks. Every trial contained two target elements (T1, T2) that replaced the distracter elements (see Figure 1). Target and distracter elements were chosen randomly without replacement. Participants were instructed to identify T1 and T2. T1 was presented as the 12th element in the stream, and T2 was presented as either the 14th element in the stream (Lag 2) or as the 17th element in the stream (Lag 5).In total, the RSVP stream consisted of 23 elements and 23 masks. In the simultaneous condition, the sound was played simultaneously with the T2 element. In the alert condition, the sound preceded T2 by 250 ms. A trial finished with the presentation of the response screen that was displayed 500 ms after the RSVP stream. Participants entered their responses via the keyboard. Responses were unspeeded and they were considered correct irrespective of order.^3^ Participants were required always to enter two letters; they were encouraged to guess if they were not sure about the correct answer. Inter-trial interval was 1,500 ms.

Each AB session consisted of a total of seven experimental blocks and one instruction block. During the instruction block, participants were shown an exemplary trial without tone at reduced speed. Thereafter they performed 20 practice trials at full speed which could either contain a tone (70%) or not (30%). In half of the trials, T2 was presented at Lag 2, in the other half at Lag 5. Of the experimental blocks, two blocks contained trials with the sound played simultaneously with T2 (simultaneous condition) and trials without additional tone (no-tone condition). Similarly, two blocks contained trials with the sound played simultaneously with the distracter element preceding T2 (alert condition) and trials without additional tone. In each of the four blocks, following Experiments 1 and 4 of Olivers and Van der Burg (2008), in 34% of trials no additional tone was played (11 trials Lag 2, six trials Lag 5), and in 66% of trials the additional tone was played (20 trials Lag 2, 10 trials Lag 5).^4^ The remaining three blocks contained Lag 2 trials of the no-tone condition (10 trials) and trials solely needed for the analysis of EEG data. In the latter trial type, T2 was always presented at Lag 5, and a tone was played at either Lag 1 or Lag 2. These trials were not included in the present analysis.^5^ Before a given block participants were informed, in the event that a tone were presented, of the temporal relationship between the tone and the second target. That is, they were either informed that the tone would be presented simultaneously with the second target, just before the second target, or considerably before the second target (“Gap” and “NoSim” blocks, see Table 1 for details). An overview of the trials contained in each block is given in Table 1. Block order was pseudo-randomized to ensure that within the first three blocks any block type would be presented only once, and to ensure that after the first three blocks, a given block type would not be followed by a block of the same type.

**Table 1.** Overview of Trial Types in the Seven Blocks of an Experimental Session

Block Condition	Simultaneous 1	Simultaneous 2	Alert 1	Alert 2	Gap 1	Gap 1	NoSim
Simultaneous	T2 Lag 2, tone Lag 2 (20)	T2 Lag 2, tone Lag 2 (20)					
Simultaneous	T2 Lag 5, tone Lag 5 (10)	T2 Lag 5, tone Lag 5 (10)					
Alert			T2 Lag 2, tone Lag 1 (20)	T2 Lag 2, tone Lag 1 (20)			
Alert			T2 Lag 5, tone Lag 4 (10)	T2 Lag 5, tone Lag 4 (10)			
No-tone	T2 Lag 2 (11)	T2 Lag 2 (11)	T2 Lag 2 (11)	T2 Lag 2 (11)	T2 Lag 2 (10)	T2 Lag 2 (10)	T2 Lag 2 (10)
No-tone	T2 Lag 5 (6)	T2 Lag 5 (6)	T2 Lag 5 (6)	T2 Lag 5 (6)			
Gap					T2 Lag 5, gap Lag 2, tone Lag 1 or 2 (30)	T2 Lag 5, gap Lag 2, tone Lag 1 or 2 (30)	
NoSim							T2 Lag 5, tone Lag 1 or 2 (40)
Sum	47	47	47	47	40	40	50

*Note*. Simultaneous blocks (Simultaneous 1, 2) contained trials of the simultaneous and no-tone conditions. Alert blocks (Alert 1, 2) contained trials of the alert and no-tone conditions. The remaining blocks (Gap 1, Gap 2, NoSim) contained trials of the no-tone condition and trials needed for the analysis of EEG data collected in parallel to behavioural data. The number of trials of a particular type is given in brackets. Trial types used for statistical analyses in the present study are highlighted in grey.

### Data analysis

#### Performance in the AB task

##### T1 performance

T1 performance was calculated for every experimental condition and T2 lag. T1 performance was statistically tested with a two-way repeated-measures ANOVA with factors Condition (no-tone, simultaneous, alert) and T2 Lag (2, 5).

##### T2 performance

T2 identification rate was calculated for trials in which T1 was identified correctly. Separate values were derived for every condition and T2 lag. Conditional T2 performance (T2|T1) was statistically tested with a two-way repeated-measures ANOVA with factors Condition (no-tone, simultaneous, alert) and Lag (2, 5). Where required Huynh-Feldt correction was applied; in these cases corrected p-values and corrected degrees of freedom are reported.

#### Reliability of AB magnitude

Previous research suggests that AB magnitude is test-retest reliable across runs and sessions and that it is also internally consistent (Dale & Arnell, 2011; Kelly & Dux, 2011). Yet findings also suggest that AB magnitudes across different versions of the AB task might not be related (Kelly & Dux, 2011). While for the present versions of the AB paradigm basic characteristics of the AB were kept constant, the simultaneous or the preceding tone might differentially affect participants. This would be reflected in reduced between-version correlations of AB magnitudes.

Reliability calculations were based on AB magnitudes. AB magnitude was defined following Martens and colleagues (Martens, Munneke, Smid, & Johnson, 2006; Martens & Valchev, 2009) as (T1Lag2 -T2|T1Lag2)/T1Lag2 × 100%. Individual AB magnitudes were screened for extreme values (i.e., outliers). An *outlier* was defined as someone whose AB magnitude exceeded the group mean AB magnitude by more than three standard deviations. This was the case for three participants. These participants were removed from the sample for all reliability calculations. For consistency, the three participants were also removed from all other statistical analyses.

To calculate split-half reliabilities, for each AB session two AB magnitudes per condition were derived. AB magnitudes were respectively based on the first half and the second half of trials for each condition. For the simultaneous condition, this corresponds to the AB magnitudes derived for the two experimental blocks containing the condition’s trials. The same applies for the alert condition. A different approach was taken for the no-tone condition, where trials were distributed across all seven blocks of an experimental session. Here, separate AB magnitudes were calculated for the first and second half of Lag 2 trials of the experimental session. In other words, for the no-tone condition, *first half* corresponds to trials presented in Blocks 1 to 3 and the first half of no-tone trials in Block 4, *second half* corresponds accordingly to the second half of no-tone trials in Block 4 and no-tone trials in Blocks 5 to 7. Pearson *r* correlations were then calculated for AB magnitudes for each condition and a Spearman-Brown correction was performed on correlations to correct for the split-half procedure (Nunnally, 1978). Pearson r correlations were also performed to estimate test-retest reliabilities. Correlations were based on AB magnitudes of the first and the second AB session for the no-tone, simultaneous, and alert conditions. The relationship between the three conditions was tested with Pearson r correlations both within and across sessions. Within-session calculations were based on the AB magnitudes used for calculating test-retest reliabilities.

To better understand the results of split-half and test-retest reliability calculations, additionally AB performance was tested for the presence of time on task effects. In detail, the factors Session (first session, second session) and Half (first half, second half) were tested in a four-way repeated measures ANOVA of conditional T2 performance (T2|T1). The remaining two factors were, as before, Lag (2, 5) and Condition (simultaneous, alert, no-tone). In parallel to the calculations of AB magnitude described above, the factor Half corresponded to performance in the first and the second half of trials in a given condition. For the simultaneous and alert conditions, this was equal to the first and the second run of the simultaneous and alert condition blocks. Because trials of the no-tone condition were distributed across all blocks, for the no-tone condition, the factor Half equalled performance in the first and the second half of trials of a session irrespective of block type (see previous paragraph for more details).

#### Questionnaires and neuropsychological tests

##### Cognitive Failures Questionnaire

For the CFQ, the distractibility score was calculated (Lumb, 1995). The distractibility score includes nine of the 32 CFQ items. Each item is rated on a 5-point scale ranging from *never* (0) to *very often* (4), thus the distractibility score ranges from 0 to 36. These items cover every-day experiences such as forgetting the location of things, getting distracted, or forgetting to transfer a message. Individual distractibility scores were compared with individual AB magnitudes from the three experimental conditions. In addition, for the alert and the simultaneous conditions, the no-tone AB magnitude was subtracted from the alert and the simultaneous AB magnitudes to derive individual gain scores. A negative gain score would reflect that a participant benefited from the tone and reduced his or her AB magnitude, a gain score around zero would indicate that the tone did not help the participant to improve performance, and a positive gain score would indicate that the tone impaired performance as compared to the no-tone condition. Gain scores were also correlated with distractibility scores.

##### Cognitive flexibility

Analysis of the NEO-FFI focused on the personality traits assumed to reflect cognitive flexibility. In detail, conscientiousness has been associated with less cognitive flexibility, while openness has been associated with more cognitive flexibility (Le Pine, Colquitt, & Erez, 2000). MacLean and Arnell (2010) studied whether personality traits could predict the AB and found that conscientiousness predicted lower overall target accuracy, while openness predicted smaller ABs. In the present study, age and sex-matched normative values (*T*-scores ^6^) were used for conscientiousness and openness as provided by Borkenau and Ostendorf (2008).

The Flexibility subtest of the TAP was used to derive laboratory-based measures of cognitive flexibility. Normative values (*T*-scores corrected for age, gender, and education as provided by Zimmermann & Fimm, 2007) were derived for speed-accuracy trade-off, median response time, errors, and the total performance index, which incorporates response time and error values (Zimmermann & Fimm, 2007).

Conscientiousness and openness scores as well as performance measures from the Flexibility subtest were correlated with individual AB magnitudes from the three experimental conditions and gain scores for the alert and the simultaneous conditions (see the *Cognitive Failures Questionnaire* section for details). Conscientiousness and openness scores were also correlated with individual AB magnitudes from the alert and the simultaneous conditions.

##### Crossmodal integration and divided attention

As recommended by Zimmermann and Fimm (2007), for the Crossmodal Integration subtest normative values (*T*-scores ) were derived for median response times. For the Divided Attention subtest (divided attention condition), normative values were derived for median response times to auditory and visual stimuli. All performance data were correlated with individual AB magnitudes from the three conditions and gain scores for the alert and the simultaneous conditions (for details, see the *Cognitive Failures Questionnaire* section).

## Results

### T1 performance and crossmodal facilitation of T2 performance

#### T1 performance

The two-way repeated measures ANOVA of T1 performance with factors Condition and Lag revealed no significant main effects of either factor: Condition, *F*(2, 40) = 0.3, *p* = .74; or Lag, *F*(1, 20) = 0.05,*p* = .83.

#### T2 performance

**Figure 2.**
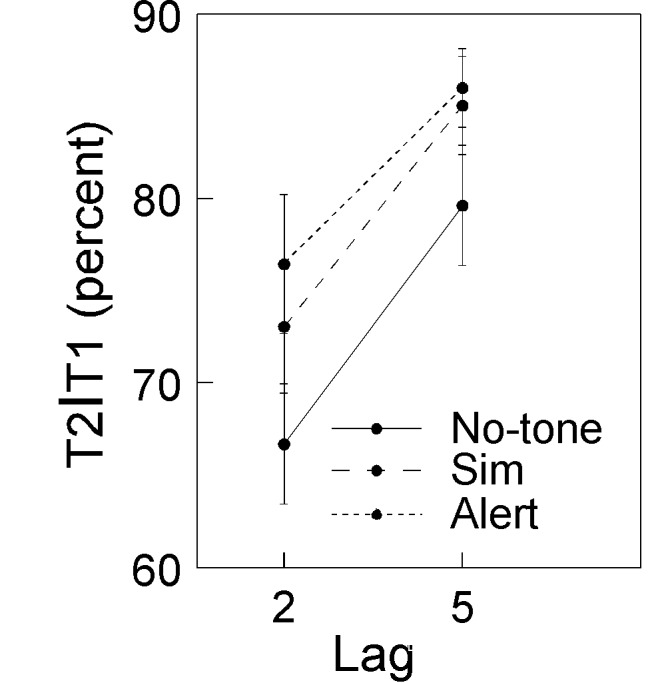
An AB was observed for all three conditions. Performance was significantly better in the alert and in the simultaneous conditions than in the no-tone condition.

An AB was observed for all conditions. Performance was overall better for alert and simultaneous conditions than for the no-tone condition (Figure 2). This pattern of results was confirmed by the repeated-measures ANOVA that revealed significant main effects of the factors Condition and Lag: Condition, *F*(2, 40) = 7.9, *p* = .001; Lag, *F*(1, 20) = 43.1, *p* < .0001. Performance at Lag 2 was significantly worse than at Lag 5 (72.1 vs. 83.6%).^7^ The main effect of condition was due to a significantly higher T2 detection rate in the alert (81.2%) and simultaneous (79.1%) conditions as compared to the no-tone condition (73.1%); *t*(20) = 3.5, *p* = .002; and *t*(20) = 2.7, *p* = .015, respectively. Simultaneous and alert conditions were however not significantly different, *t*(20) = 1.3, *p* = .21.

### Reliability of AB magnitudes

#### Split-half reliability of AB magnitudes

Session-wise split-half reliabilities were of mixed size. In Session 1, it was found (but moderate) for all three conditions, with correlations of *r* = .46, *r* = .40,^8^ and *r* = .44 (cf. Table 2). For Session 2, all correlations were small. Only for the no-tone condition did the correlation reach significance after Spearman-Brown correction (Spearman-Brown corrected *r* = .53, *p* < .05).

**Table 2.** Split-Half Reliability of AB Magnitude

	*r*	Spearman-Browncorrected *r*
No-tone condition, Session 1	.46*	.63*
No-tone condition, Session 2	.36	.53*
Simultaneous condition, Session 1	.40(*)	.57*
Simultaneous condition, Session 2	.03	.08
Alert condition, Session 1	.44*	.61*
Alert condition, Session 2	.15	.26

*Note*. (*)*p* < .10. *p < .05.

#### Test-retest reliability of AB magnitudes

**Figure 3.**
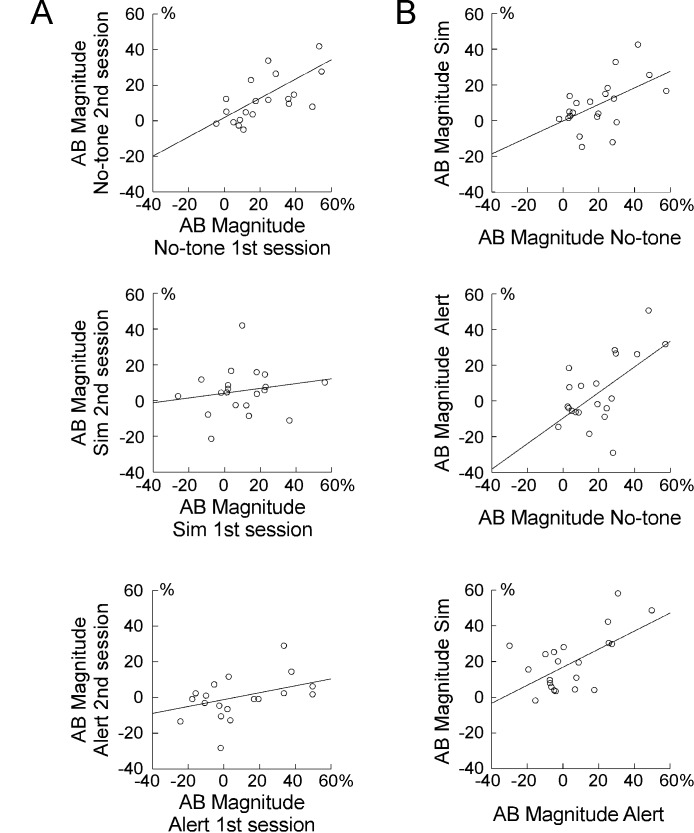
Panel A. Scatter plot depicting test-retest reliability for the no-tone condition (top), the simultaneous condition (middle), and the alert condition (bottom). Panel B. Scatter plot depicting Pearson *r* correlations for simultaneous and no-tone conditions (top), for alert and no-tone conditions (middle), and for simultaneous and alert conditions (bottom).

Test-retest reliability was shown for the no-tone and alert conditions (cf. Panel A of Figure 3). Pearson correlations between the first and second session were *r* = .73 (*p* < .001) for the no-tone and *r* = .45 (*p* < .05) for the alert condition. For the simultaneous condition, test-retest reliability was not confirmed (*r* = .22, *p* = .32).

#### Relationship between conditions

No-tone and simultaneous conditions correlated significantly (*r* = .53, *p* = .01), as did no-tone and alert conditions (*r* = .60, *p* < .01). For simultaneous and alert conditions only a significant trend was observed (*r* = .42, *p* = .06; see Panel B of Figure 3). As shown in Table 3, correlations were very comparable if considered separately for each session.

**Table 3.** Within-Session Pearson *r* Correlations Across Conditions’ AB Magnitudes

*r*	
No-tone condition, Session 1, and Simultaneous condition, Session 1	.48*
	
No-tone condition, Session 1, and Alert condition, Session 1	.49*
Simultaneous condition, Session 1, and Alert condition, Session 1	.21
No-tone condition, Session 2, and Simultaneous condition, Session 2	.52*
No-tone condition, Session 2, and Alert condition, Session 2	.69**
Simultaneous condition, Session 2, and Alert condition, Session 2	.43*

*Note*. (*)*p* < .10. *p < .05. **p < .01.

#### AB performance as a function of time on task

As reliability of AB magnitudes was not found throughout, AB performance was explored for effects of time on task. In addition to the main effects of condition and lag observed for T2 performance (see the *T2 Performance* section) main effects of session, *F*(1, 20) = 27.3, *p* < .0001, and half, *F*(1, 20) = 15.0, *p* = .001, were evident. Performance increased from first to second session (73.1 vs. 82.5%) and from first to second half (75.2 vs. 80.4%), indicating the presence of time-on-task effects. Main effects were further supplemented by the significant interactions of Session × Half, *F*(1, 20) = 14.7, *p* < .01, and of Condition × Half × Lag, *F*(1.8, 36.2) = 3.5, *p* = .047.

The interaction of the factors Session and Half was followed-up by *t*-tests that indicated that whereas in the first session performance improved significantly from first to second half, *t*(20) = -5.8, *p* < .0001, the improvement in performance from first to second half was not significant for the second session, *t*(20) = -0.8, *p* = .40.

**Figure 4.**
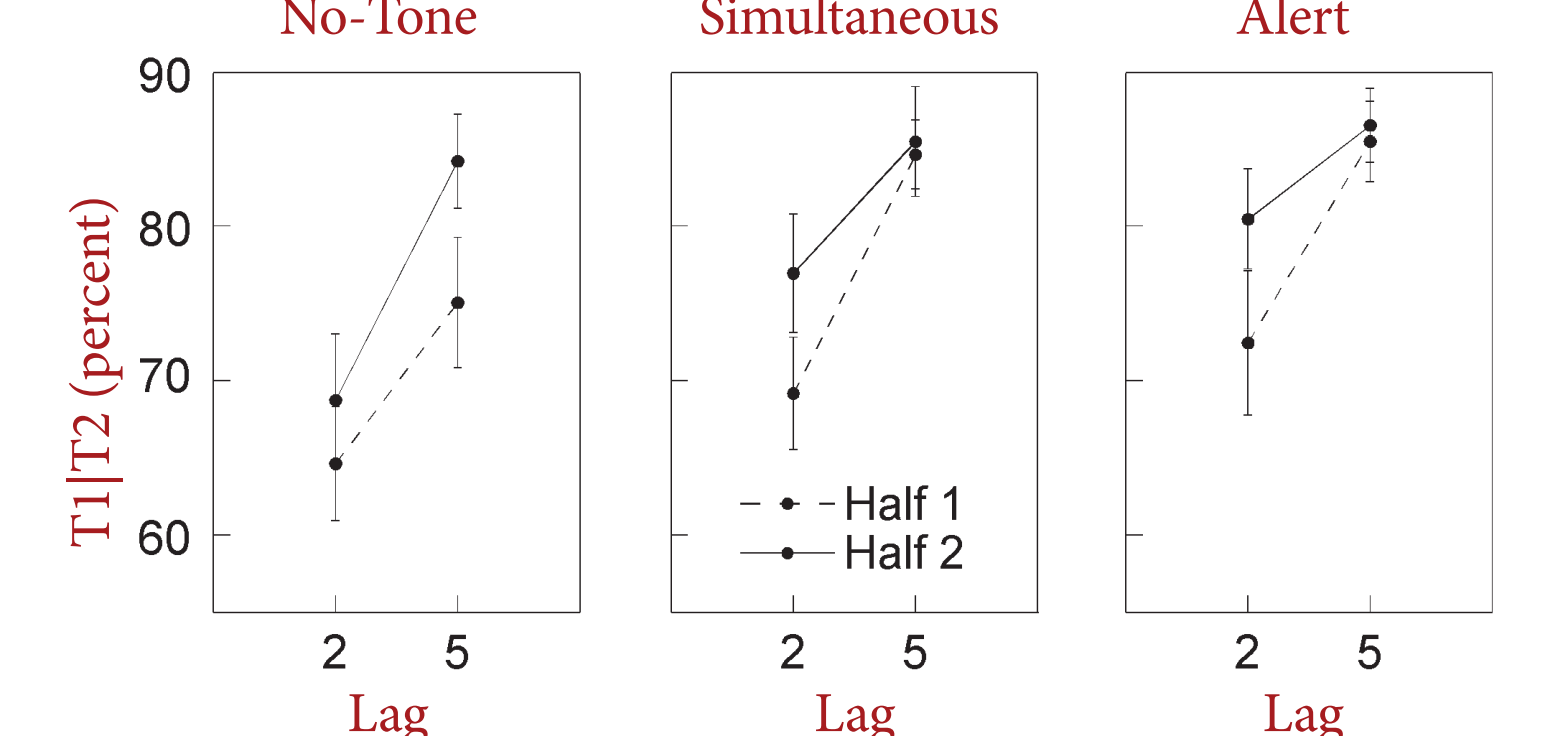
T2 performance as a function of lag. In the no-tone condition, performance improved from first to second session irrespective of lag. In the simultaneous condition, practice improved performance at Lag 2 but not at Lag 5, effectively resulting in a reduction of the AB. Similarly, in the alert condition practice improved T2 performance at Lag 2 but not at Lag 5, thereby reducing the AB. For the Alert condition this effect was not signifcant though.

The three-way interaction Condition × Half × Lag was followed by condition-specific two-way ANOVAs with factors Half and Lag. For the no-tone condition, this ANOVA indicated main effects of half, *F*(1, 20) = 8.4, *p* < .01, and lag, *F*(1, 20) = 39.1, *p* < .0001, but no inter-action, *F*(1, 20) = 1.6, *p* = .20. That is, T2 performance was significantly better at Lag 5 than at Lag 2, and irrespective of lag significantly improved from first to second half (see Figure 4, left panel). For the simultaneous condition, the results of the two-way ANOVA indicated main effects of lag, *F*(1, 20)= 40.4, *p* < .0001, and half, *F*(1, 20) = 5.1, *p* < .05, as well as a significant interaction, *F*(1, 20) = 5.5, *p* < .05. Subsequent *t*-tests revealed that both halves showed a significant AB; first half Lag 2 versus Lag 5: *t*(20) = -6.4, *p* < .0001; second half Lag 2 versus Lag 5: *t*(20) = -3.6, *p* < .01. Nevertheless, the improvement from the first to second half was only evident for Lag 2, *t*(20) = -3.7, *p* < .001, not for Lag 5, *t*(20) = -0.31, *p* = .76. In other words, the simultaneous tone apparently helped to improve performance with practice at Lag 2 but not at Lag 5, effectively resulting in a time-on-task-related reduction of the AB. This reduction of the AB was statistically confirmed by the significant result of a *t*-test comparing the difference between Lag 5 and Lag 2 performance for the first and second half, *t*(20) = 2.4, *p* < .05. As is illustrated in Figure 4 (middle panel), the reduction of the difference between Lag 5 and Lag 2 was entirely due to improved performance at Lag 2. It was then tested whether this practice-related reduction of the AB might affect any condition-related variations of the AB. To address this question we compared the difference between Lag 5 and Lag 2performance for the no-tone and for the simultaneous conditions in a post-hoc *t*-test. The result of this test indicated that the Lag 5 - Lag 2 difference was significantly smaller in the simultaneous condition for the second half, *t*(20) = -3.1, *p* < .01, but not the first half, *t*(20) = 0.8, *p* = .40.

Visual inspection of Figure 4 (right panel) indicated that the time-on-task effects for the alert condition were very comparable to the simultaneous condition. Accordingly, the main effects of lag, *F*(1, 20) = 11.8, *p* < .01, and half, *F*(1, 20) = 5.4, *p* < .05, were significant. However, the interaction did not reach significance, *F*(1, 20) = 3.4, *p* = .08.

### AB magnitudes, tests of attentional performance, and questionnaires

#### Cognitive Failures Questionnaire (CFQ)

The CFQ distractibility score ranged between 6 and 19, and was positively correlated with the simultaneous condition gain score (*r* = .43, *p* < .05). That is, people who described themselves as rather distractible did not benefit from the simultaneous tone. No other significant correlations were observed (all *rs* < .30).

#### Cognitive Flexibility

Neither openness (range of *T*-scores : 22-61) nor conscientiousness (range of *T*-scores : 41-78) of the NEO-FFI correlated significantly with AB magnitudes or the gain scores of the alert and simultaneous conditions (all *rs* between -.24 and .24).

No significant correlations were observed for no-tone and simultaneous conditions, AB magnitudes, or the gain scores of the alert and the simultaneous conditions with any of the assessed measures of the Flexibility subtest (all *rs* between -.42 and .22; range of *T*-scores speed-accuracy trade-off = 41-70, median response time = 41-63, errors = 42-61, total performance index = 41-61). The number of errors and the speed-accuracy trade off however correlated with AB magnitude derived for the alert condition. A small AB magnitude was related to a low error rate (*r* = -.545, *p* = .011) and a small speed-accuracy trade off (*r* = -.441, *p* = .046).

#### TAP crossmodal integration and divided attention

Median response time *T*-scores for the Crossmodal Integration subtest ranged between 33 and 59. For the Divided Attention subtest, *T*-scores ranged between 31 and 63 for median response times to auditory stimuli, and between 45 and 66 for median response times to visual stimuli. No significant correlations were observed for the Crossmodal Integration subtest, similarly for Divided Attention subtest and the No-tone AB magnitude, as well as the gain scores of the alert and the simultaneous conditions (all *rs* between -.41 and .43). AB magnitudes of the no-tone and the alert conditions however correlated with the median response times to visual stimuli in the divided attention setup. Small AB magnitudes were related to long response times (no-tone condition: *r* = .51, *p* = .018; alert condition: *r* = .441, *p* = .046; all other: *rs* between -.32 and .24).

## Discussion

The present study aimed to investigate the effect of a sound on T2 identification in an AB paradigm. Three experimental conditions were studied. In the simultaneous condition, the tone was played simultaneously with T2; in the alert condition, the tone preceded T2 by 250 ms; and in the no-tone condition, no additional tone was played. For all studied experimental conditions an AB was observed. T2 performance was significantly improved in the simultaneous and alert conditions. AB magnitudes obtained for the three conditions were split-half reliable only for the first of two experimental sessions, and test-retest reliability was only found for the no-tone condition. AB magnitude of the no-tone condition correlated significantly with AB magnitudes of the simultaneous and alert conditions. Whether participants benefited from the simultaneous tone was related to their self-reported everyday distractibility. Good performance in a test of cognitive flexibility was linked to a small AB magnitude in the alert condition.

### The effect of a tone on target identification

The simultaneous tone improved T2 detection rate irrespective of target-to-target SOA. This is in good agreement with previously reported results (Olivers & Van der Burg, 2008). However, if the tone was played 250 ms before T2, T2 identification improved at least as much as in the simultaneous condition, which is in direct contrast to the findings by Olivers and Van der Burg. Because Olivers and Van der Burg did not observe any effect of the alerting sound they concluded that alerting plays no role in the improvement of T2 performance by a simultaneous sound. Our results for the alert condition challenge this conclusion and indicate that the sound does raise alertness and leads to an attentional enhancement. In spite of this, our data do not allow us to differentiate between whether improved T2 identification in the simultaneous condition is due to mechanisms that arise independently of alertness as suggested by Olivers and Van der Burg, whether it is due to alertness, or a combination of both.

Our results are in line with the findings by Van Vleet and Robertson (2006), who tested a visual neglect patient in an AB paradigm. In this study, if T2 was preceded by a sound, T2 performance improved. Olivers and Van der Burg (2008) suggested that the difference between their findings and those of Van Vleet and Robertson (2006) indicates that whereas for the neglect patient the sound was an effective alerting signal that increased arousal, it was not effective for their sample of healthy participants, likely because their level of arousal was high to begin with. According to this interpretation, our results would indicate that our participants’ overall level of arousal was rather low, and that it could therefore be temporarily raised by the alerting sound. Yet our participants were young and healthy students, just as in the study by Olivers and Van der Burg. Thus, it is difficult to see why our participants’ overall arousal level should be considerably reduced and be responsible for the different findings in the present study and in the study by Olivers and Van der Burg.

A more likely reason for the fact that, in contrast to Olivers and Van der Burg (2008), we did find significant effects of an alerting sound on T2 identification, could be differences in the experimental design. Even though we kept the ratio of trials with a tone and of trials without a tone close to Experiment 2 of Olivers and Van der Burg, in our experiment, in the blocks that contained the alert condition trials, the tone would always precede T2 but never T1. The alerting tone is therefore 100% valid as to the timing of T2. This contrast to the original study, where the tone was presented at three different time points relative to T2, could be responsible for the observed effect of the alerting tone. It is interesting to note that in the study by Van Vleet and Robertson (2006), where the tone was also found to be alerting, the design similarly incorporated a 100% validity of the alerting tone for T2. Moreover, the observation that an alerting sound can improve T2 identification also fits well into the body of research on crossmodal cueing (Bertelson & Tisseyre, 1967; Chen & Spence, 2011; Los & van den Heuvel, 2001; McDonald, Teder-Salejarvi, & Hillyard, 2000; Ngo & Spence, 2010; Niemi & Näätänen, 1981). Moreover, a recent study that used a within-modality manipulation to induce a phasic increase in alertness also reports findings that support the present results of an alerting effect of the tone (Spalek & Di Lollo, 2011).

It is conceivable that other differences in experimental design also contributed to the differences in the present findings and those of Olivers and Van der Burg (2008). One such could be the difference in presentation duration of target and distracter elements in the present study and in the study by Olivers and Van der Burg. The performance data argue against this possibility in that they are descriptively very comparable in each study, and that differences were only observed in one of three conditions, though stimulus presentation duration was identically changed in all three. Another relevant difference might be different task instructions. Olivers and Van der Burg’s participants were told that “sound may accompany targets” (p. 198), and thus had to figure out the nature of the relationship between sound and target themselves. In our experiment, before each block, participants were informed about the to-be-expected temporal relationship of tone and T2 when a tone was present. This might have been particularly helpful for making use of the alerting tone. However, the evidence at hand does not yet allow us to draw concrete conclusions with regard to this or any other of the discussed differences. Only a systematic within-study comparison will be able to do so.

### Reliability of AB magnitudes and correlations

The results of our study regarding the reliability of AB magnitudes are rather mixed. While in the first session split-half reliability was mode-rate but found irrespective of whether a tone was presented or not, it was not observed in the second session for the alert and simultaneous conditions. For the no-tone condition, only the Spearman-Brown corrected *r* reached significance in the second session. Test-retest re-liability was shown for the no-tone condition, which is in agreement to previous research (Dale & Arnell, 2011). The simultaneous and alert conditions were not test-retest reliable though. The pattern of results suggests that in certain cases AB magnitude is susceptible to state factors. For the present study it is conceivable that in the second session participants knew what to expect. Based on their experience in the first session, they started out with a strategy they hoped would help them with the task, in particular in trials when a tone was presented. If the strategy did not work they would drop it in the course of Session 2, maybe returning to what they did in Session 1 or trying a different strategy. Both would affect split-half reliability and test-retest reliability.

We did find significant correlations between the no-tone and simultaneous and the no-tone and alert conditions. This shows that in spite of its susceptibility to state factors, AB magnitude also reflects dispositional ability or style, as suggested by Dale and Arnell (2011). For the simultaneous and alert conditions, the correlation did not quite reach significance. Thus, besides joint factors, separate factors seem to affect performance in these two conditions and determine the degree of benefit from an alerting or a simultaneous tone.

The conclusion that performance in the alert and simultaneous conditions is influenced by joint and separate factors is supported by the observation that they correlated differently with neuropsychological scores. In detail, response times to visual stimuli in a divided attention setup were negatively linked to AB magnitudes of alert and no-tone conditions only. Though surprising at first glance, the negative relationship is in line with previous research reporting that an overall low information processing speed is linked to small AB magnitudes (Visser & Ohan, 2012; but see e.g., Arnell, Howe, Joanisse, & Klein, 2006). Visser and Ohan suggest that this pattern might reflect that slow information processers have fewer resources available to process distracters and are therefore less impaired in target processing in the AB task, in particular when targets and distracters are highly similar. Differences were also evident for a test of cognitive flexibility. Here, good performance was linked to small AB magnitudes in the alert but not in the simultaneous condition. This could reflect that individuals with higher cognitive flexibility can more easily disengage from the alerting tone and then re-engage in time to the target. Such ability would however not help in the simultaneous condition. Finally, a high proneness to every-day distractibility was linked to a lack of gain from the simultaneous condition, whereas there was no link to the alert condition. This indicates that while in the simultaneous condition distraction by the tone and distractibility of the individual are factors to take into consideration when assessing performance, this is not the case in the alert condition.

In contrast to our expectations, we did not observe a correlation of behavioural measures of the AB with conscientiousness and openness, personality dimensions that have been related to performance in the AB task (MacLean & Arnell, 2010). Closer inspection of the findings by MacLean and Arnell indicates however that, in particular for openness, observation of a relationship heavily depended on the analysis being performed in a regression that included four of the five NEO-FFI dimensions (neuroticism, extraversion, openness, and conscientiousness), rather than in a bivariate correlation. Unfortunately, MacLean and Arnell did not comment on what this might mean from a theoretical point of view. One possibility would be that in the regression analysis, one of the additional variables acted as a suppressor variable (Bortz, 2005). Investigating this possibility and potential alternatives is beyond the scope of the present study, but it would be an interesting focus of future work.

### Combined effect of practice and sound on visual task performance

An unexpected result was the finding that the effect of the sound on T2 performance and the effect of practice interact, in particular if the sound is presented simultaneously with T2. Thus, it appears that there is an immediate but general effect of the sound on T2 identification and, in addition, a time-on-task or practice effect on T2 identification. But it is the combination of these mechanisms that specifically improves the identification of T2 items presented at short target-to-target SOA, and thus specifically reduces the AB. This interaction can be interpreted in two ways: The sound might speed-up the purely practice-related reduction of the AB, which would normally emerge only after a much larger amount of practice (Maki & Padmanabhan, 1994) or with more closely spaced experimental sessions (Nakatani, Baijal, & van Leeuwen, 2012) and therefore is not evident in the no-tone condition of the present study. Alternatively, practice might boost the automatic effec*t*(s) of the sound on T2 identification. That is, participants might learn to make better use of the sound. Yet as performance is already at ceiling if T2 is presented with a long target-to-target SOA and is either preceded or accompanied by a tone, this will only affect T2 presented with a short target-to-target SOA. In consequence, the AB becomes smaller. Unfortunately, at present, our data do not allow us to favour either interpretation.

The impact that a non-specific sound can exert on visual target identification and vice versa has been the focus of several recent studies (Kim, Peters, & Shams, 2012; Olivers & Van der Burg, 2008; Thorne & Debener, 2008; Van der Burg, Olivers, Bronkhorst, & Theeuwes, 2008; Van der Burg, Talsma, Olivers, Hickey, & Theeuwes, 2011; Vroomen & Gelder, 2000). Results appear to be comparable for the combination of different modalities, such as a tactile task and a non-specific sound (Van der Burg, Olivers, Bronkhorst, & Theeuwes, 2009). All studies report that the different-modality stimulus improves performance, and all studies see this effect as automatic and basically in-built. That is, the perceptual representation of the target stimulus is assumed to benefit from the non-specific, different-modality stimulus from the first combined presentation of sound and target. Our data challenge this assumption as they indicate that cross-modal facilitation needs some time to become most effective, but more research is clearly needed to substantiate this idea.

### Implications for theories of the attentional blink

T2 performance improved with the presentation of a preceding or a simultaneous tone. However, there was no evidence of a trade-off between improvements in T2 performance and T1 performance. This is inconsistent with traditional limited-capacity accounts of the AB, which basically propose that there are a limited amount of processing resources that have to be shared between the two targets (for a review, see Dux & Marois, 2009). Based on this idea the prediction would be that a shift of resource distribution to either target would affect processing of the other target. The pattern of results is however in agreement with the idea that an overinvestment of resources to the processing of T1 and/or the distracter stream contributes to the AB (see e.g., Kranczioch, Debener, Maye, & Engel, 2007; Olivers & Nieuwenhuis, 2005, 2006; Shapiro, Schmitz, Martens, Hommel, & Schnitzler, 2006; Slagter et al., 2007). If this mechanism can be overridden, T1 performance will be unaffected but T2 performance will improve. Apparently, a tone, no matter whether it is presented before T2 or simultaneously to it, serves this purpose well.

With regard to accounts that explain the AB as being due to inhibition triggered by the post-T1 distracter (Di Lollo, Kawahara, Shahab Ghorashi, & Enns, 2005; Olivers & Meeter, 2008) our results are not conclusive. The simultaneous tone apparently strengthened the representation of T2. If one assumes that this occurs more or less automatically, then it is likely that the alerting tone, which was presented simultaneously with the post-T1 distracter, similarly strengthened the post-T1 distracter. A strong post-T1 distracter should result in strong inhibition of subsequent stimuli and a large AB. The present results however show the opposite. A retort by the post-T1 distracter accounts would be to suggest that due to the present experimental design inhibition was already triggered by the mask that followed T1 and that preceded the post-T1 distracter. In this scenario, the post-T1 distracter would already be inhibited. Inhibition might also delay the effect of the tone, or suppress its immediate consequences on the post-T1 distracter while leaving its alerting properties to take effect on T2.

## Summary and conclusions

The present results show that in the AB paradigm, an alerting tone can improve the identification of T2, in the same way as a simultaneous tone. This indicates that even though the tone might automatically facilitate visual-perceptual processes in this paradigm, it can also facilitate target processing by alertness and a general attentional enhancement. Additional analyses indicated that in face of the overall tone-related improvement of T2 performance, AB magnitudes were reliable if compared to the no-tone condition. Thus, performance in all three conditions studied was affected by a dispositional ability or style. Individual differences might however exist in how a person is affected by the simultaneous tone as compared to the alerting tone. Mixed results for split-half and test-retest reliabilities suggest that in addition to dispositional abilities state factors can also exert an influence on AB magnitude. In addition to the tone-related overall improvement of T2 identification, a reduction of the AB was observed when taking into consideration time-on-task. This reduction was only present when a tone accompanied T2 and was strongest when the tone occurred simultaneously with T2. This new finding indicates that audition-driven facilitation of visual information processing is not entirely automatic, but may become more effective after the repeated exposure to the auditory-visual stimulus combination. More research is needed to follow up this idea.

## Footnotes

1 In this study, the presentation rate for targets and distracters was 4 per second. In consequence, the Lag 2 T2 was presented at a time where in a typical AB paradigm the AB has largely recovered. The authors showed that in spite of this T2 identification performance was substantially impaired at short T2 lags as compared to long T2 lags. This is seen as the primary indicator of the AB effect (MacLean & Arnell, 2012).

2 Note that this is a change as compared to the study by Olivers and Van der Burg (2008) who presented their stimuli for 75 ms, followed by a 25-ms break. This adaptation was necessary to bring the performance level in the no-tone condition (Lag 2) in the range of the performance level reported by Olivers and Van der Burg.

3 Overall, order reversals in trials in which both T1 and T2 were correctly identified were less than 5%. For Lag 2 it was 4.6% for the simultaneous condition, 4.3% for the no-tone condition, and 3.2% for the alert condition. For Lag 7 it was 2.8% for the simultaneous condition, 3.1% for the no-tone condition, and 2.4% for the alert condition.

4 Throughout the experiment and for all three experimental conditions frequencies of Lag 2 trials were higher than those of Lag 5 trials to allow for a performance-based analysis of physiological data collected for Lag 2 trials.

5 Paired *t*-tests showed that performance in these trials was not significantly different from performance in the Lag 5 no-tone trials (all ps > .14).

6 *T*-scores are standardized values with a mean of 50 and standard deviation of 10. Values below 40/above 60 are considered low/high ave-rage; values below 30/above 70 are considered as borderline/superior (Lezak, Howieson, & Loring, 2004).

7 If considered separately for condition, performance in the alert condition was 76.4% for Lag 2 and 86.0% for Lag 5, in the simultaneous condition: 73.1% for Lag 2 and 85.1% for Lag 5, and in the no-tone condition: 66.7% for Lag 2 and 79.6% for Lag 5.

8 p < .10, Spearman-Brown corrected *r* = .57, *p* < .05.
